# Histiocitosis de células de Langerhans diagnosticada en ganglios mediastínicos mediante punción-aspiración con aguja fina guiada por ecobroncoscopia

**DOI:** 10.23938/ASSN.1137

**Published:** 2025-12-04

**Authors:** Tania Labiano Miravalles, Ana Echegoyen, Andreina Stefanía De Oliveira, Begoña Álvarez, Sergio Curi, Elena Almudévar

**Affiliations:** 1 Servicio Navarro de Salud-Osasunbidea Hospital Universitario de Navarra Servicio de Anatomía Patológica Pamplona España; 2 Servicio Navarro de Salud-Osasunbidea Hospital Universitario de Navarra Servicio de Radiología Pamplona España; 3 Servicio Navarro de Salud-Osasunbidea Hospital Universitario de Navarra Servicio de Neumología Pamplona España

**Keywords:** Biopsia por Aspiración con Aguja Fina Guiada por Ultrasonido Endoscópico, Citología, Histiocitosis de Células de Langerhans, Mediastino, Broncoscopia, Endoscopic Ultrasound-Guided Fine Needle Aspiration, Histiocytosis, Langerhans-Cell, Mediastinum, Cytological Techniques, Bronchoscopy

## Abstract

Varón de 57 años de edad, fumador, al que se le realiza punción aspiración con aguja fina guiada por ecobroncoscopia por presentar adenopatías hipercaptantes en la tomografía por emisión de positrones. La citopatóloga procesó *in situ* extensiones citológicas para evaluación rápida (Diff-Quick) y diferida (Papanicolaou). Con los fragmentos sólidos y los coágulos obtenidos realizó un bloque celular que se procesó como biopsia.

En las extensiones se observaron células de aspecto histiocitoide con núcleos hendidos, inmunorreactivas para CD68, CD1a, Langerina y S100, con abundantes eosinófilos; el diagnóstico final fue de histiocitosis de células de Langerhans. El diagnóstico citológico preciso de esta entidad en ganglios mediastínicos supone un gran reto. Este caso queremos resaltar que un buen manejo del material por el citopatólogo presente en la sala es clave para rentabilizar el tejido y poder realizar las técnicas complementarias necesarias para un diagnóstico completo, evitando abordajes diagnósticos más agresivos.

## Abstract (en)

A 57-year-old male smoker underwent endobronchial ultrasound-guided fine-needle aspiration due to increased radiotracer uptake on positron emission tomography. The cytopathologist prepared *in situ* smears for immediate (Diff-Quick) and definitive (Papanicolaou) evaluation. Solid tissue fragments and blood clots were used to create a cell block processed as a biopsy.

Cytological analysis revealed histiocytic cells with clefted nuclei expressing CD68, CD1a, Langerin, and S100, along with a numerous eosinophils, establishing the diagnosis of Langerhans cell histiocytosis. Accurate cytological identification of this entity in mediastinal lymph nodes remains a significant diagnostic challenge. This case underscores the importance of the cytopathologist’s active involvement in sample handling during the procedure to optimize material performance, enabling the use of ancillary techniques required for a comprehensive diagnosis, avoiding more aggressive diagnostic approaches.

## INTRODUCCIÓN

La histiocitosis de células de Langerhans (HCL) es una enfermedad rara de proliferación de células del sistema fagocítico mononuclear con función aberrante. Aunque estas células se denominan con el antiguo término *histiocito* (célula tisular), hoy en día se sabe que corresponden a un determinado tipo de células del sistema fagocítico mononuclear, las células de Langerhans^1^. Se caracterizan por expresar CD1a, Langerina, S100 y, ultraestructuralmente, por presentar gránulos de Birbeck. Aunque pueden llegar a acumularse en prácticamente cualquier órgano, muestran una afinidad particular por el hueso, la piel, los pulmones y la hipófisis.

En la infancia, la edad de aparición de esta enfermedad es entre el año y los tres años, aunque también aparece en adultos, siendo más frecuente en varones^2^. La incidencia anual de la HCL en menores de 15 años es de 4,6 casos por millón, con una relación hombre-mujer de 1,2:1. Sin embargo, la incidencia estimada entre personas adultas es mucho menor, de 1 a 2 casos por millón, aunque es probable que esté infradiagnosticada^1^^,^^3^^,^^4^.

La presentación clínica de la HCL es muy variable, con síntomas que varían dependiendo del órgano afectado y que comprenden desde lesiones aisladas de curso indolente hasta una enfermedad multisistémica de curso rápido, que puede ser incluso grave cuando afecta a niños pequeños y/o cuando hay afectación de órganos vitales como sistema nervioso central, médula ósea o hígado^1^.

A continuación, presentamos un caso de HCL con afectación de ganglios linfáticos mediastínicos en un varón adulto, con el objetivo de aportar nuestra experiencia en el diagnóstico diferencial de esta enfermedad, haciendo énfasis en el diagnóstico citológico, mínimamente invasivo.

## CASO CLÍNICO

Varón de 58 años de edad, con antecedente de tabaquismo activo y gammapatía monoclonal de significado indeterminado diagnosticada a principios de 2022, a raíz de un episodio de clínica de artritis poliarticular con predominio de manos, carpos y pies.

En septiembre de 2022, en un control de la artritis y del dolor poliarticular persistente, se le realizó una radiología simple de tórax en la que, como hallazgo incidental, se observó que ambos hilios pulmonares estaban bien definidos. Por ello se amplió el estudio con una tomografía computarizada (TC) toraco-abdominal realizada en noviembre de 2022, donde se observó un notable aumento en número y tamaño de las adenopatías mediastínicas e hiliares bilaterales y, además, un nódulo con características de vidrio deslustrado de 8mm de diámetro en el lóbulo medio del pulmón derecho. En las analíticas realizadas en esta fecha se detectó elevación de la enzima convertidora de angiotensina (ECA: 87 µg/L; rango normal: <40 µg/L) pero QuantiFERON®-TB Gold negativo, sugiriendo la ausencia de infección activa de tuberculosis.

**Figura 1:**
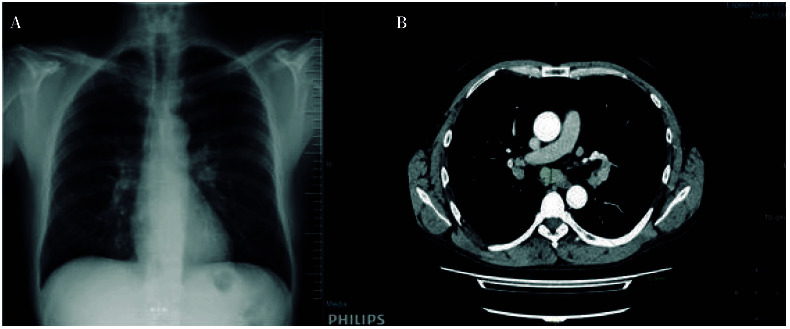
Hallazgos radiológicos en tórax. **A.** Placa simple. Se aprecia aumento de densidad pseudonodular en hilio izquierdo, sin hallazgos significativos en hilio derecho. **B**. Tomografía computarizada con contraste intravenoso. Se observan varias adenopatías hiliares bilaterales y una adenopatía subcarinal de 15 mm de diámetro menor, que fue biopsiada por punción aspiración con aguja fina a través de ecobroncoscopia guiada por ultrasonidos.

Se decidió seguimiento de un año y se repitió el estudio de TC, observándose un ligero aumento en el tamaño de las adenopatías (Fig. 1A), por lo que en octubre de 2023 se realizó una tomografía por emisión de positrones (PET) que mostró hipercaptación del radiofármaco (^18^F-FDG) en la adenopatías (Fig. 1B). La principal sospecha clínica fue de proceso granulomatoso crónico tipo sarcoidosis. Se decidió realizar una punción aspiración con aguja fina a través de ecobroncoscopia guiada por ultrasonidos (PAAF-EBUS) de las adenopatías mediastínicas ubicadas en las regiones subcarinal y paratraqueal derecha o 4R.

Durante la obtención de la muestra se administraron al paciente 6 mg de midazolam y 150 µg de fentanilo; el procedimiento se realizó sin complicaciones. Una citopatóloga estuvo presente en la sala durante la obtención de la muestra, realizando extensiones del material obtenido mediante PAAF-EBUS. Parte de las extensiones se tiñeron con Diffquick para una evaluación rápida en la sala; el resto se fijaron en etanol 96º para teñirlas con Papanicolaou posteriormente y realizar el estudio diferido. Además, se realizó un bloque celular a partir de los coágulos y fragmentos sólidos obtenidos. Al finalizar, el paciente permaneció dos horas en la sala de reanimación, sin presentar incidencias.

**Figura 2:**
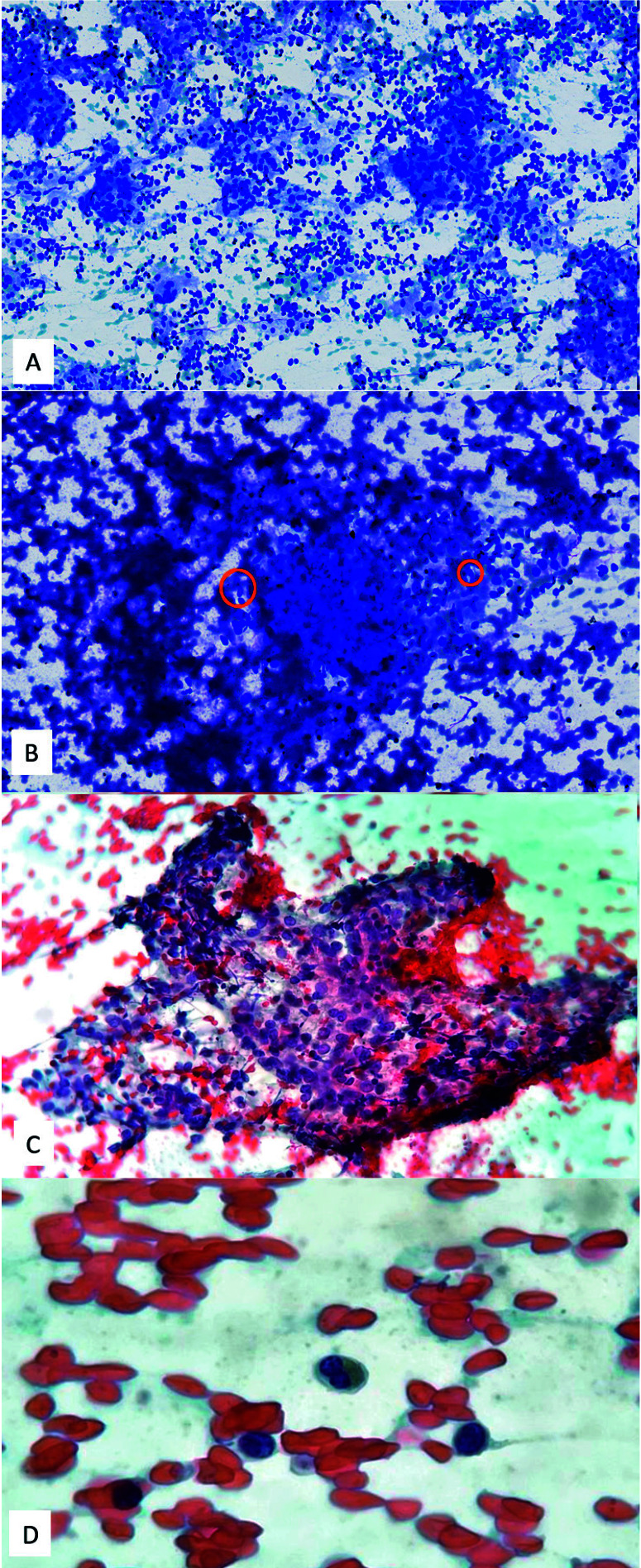
Microscopía óptica. Las extensiones teñidas con Diffquick en la sala muestran un fondo de linfocitos pequeños formando agregados (**A**) y agregados de células histiocíticas con núcleos arriñonados (círculos) (**B**). Las extensiones fijadas y teñidas con Papanicolaou muestran abundantes linfocitos sobre un fondo hemático (**C**) y ocasionales eosinófilos (D). A: 50x, B: 200x, C: 400x, D. 1000x.

Las extensiones citológicas del material obtenido mostraron un fondo moderadamente hemático con abundante población linfoide de aspecto polimorfo y, de forma ocasional, se identificaron eosinófilos. Se identificaron acúmulos linfoides con macrófagos, algunos con aspecto epitelioide y formando agregados. Los núcleos de los macrófagos presentaban barras y hendiduras con formas arriñonadas (Fig. 2).

**Figura 3:**
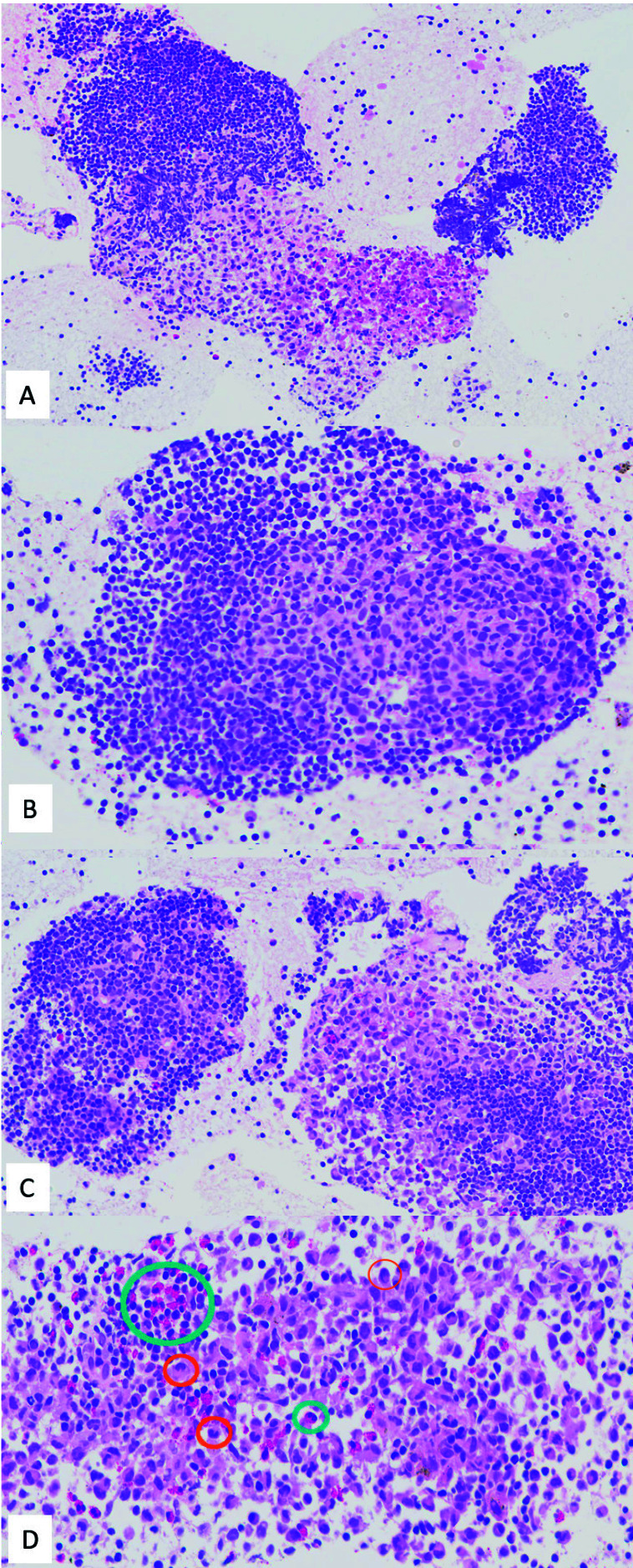
Microscopía óptica. Los cortes del bloque celular teñidos con hematoxilina-eosina muestran agregados de linfocitos con centro germinal (**B**) y agregados de células histiocíticas con citoplasma amplio y eosinofílico (**A,C,D**) y el característico núcleo arriñonado hendido (círculo naranja). Se observan eosinófilos, con un citoplasma granular intensamente eosinófilo (círculo verde), de forma dispersa o en agregados (**A**). A,C: 200x, B: 400x, D: 600x.

El bloque celular con el material sólido y los coágulos se fijó en formol y se incluyó en parafina para su estudio histológico. En los cortes teñidos con hematoxilina-eosina (Fig. 3) se observaron folículos linfoides con centros germinales reactivos, y agregados inmaduros de células de aspecto histiocitoide con citoplasmas amplios, con núcleos hendidos, de membrana nuclear irregular, con aspecto arriñonado o polilobulado, con cromatina fina. Se observaron eosinófilos entremezclados. No se identificaron granulomas no necrotizantes.

**Figura 4:**
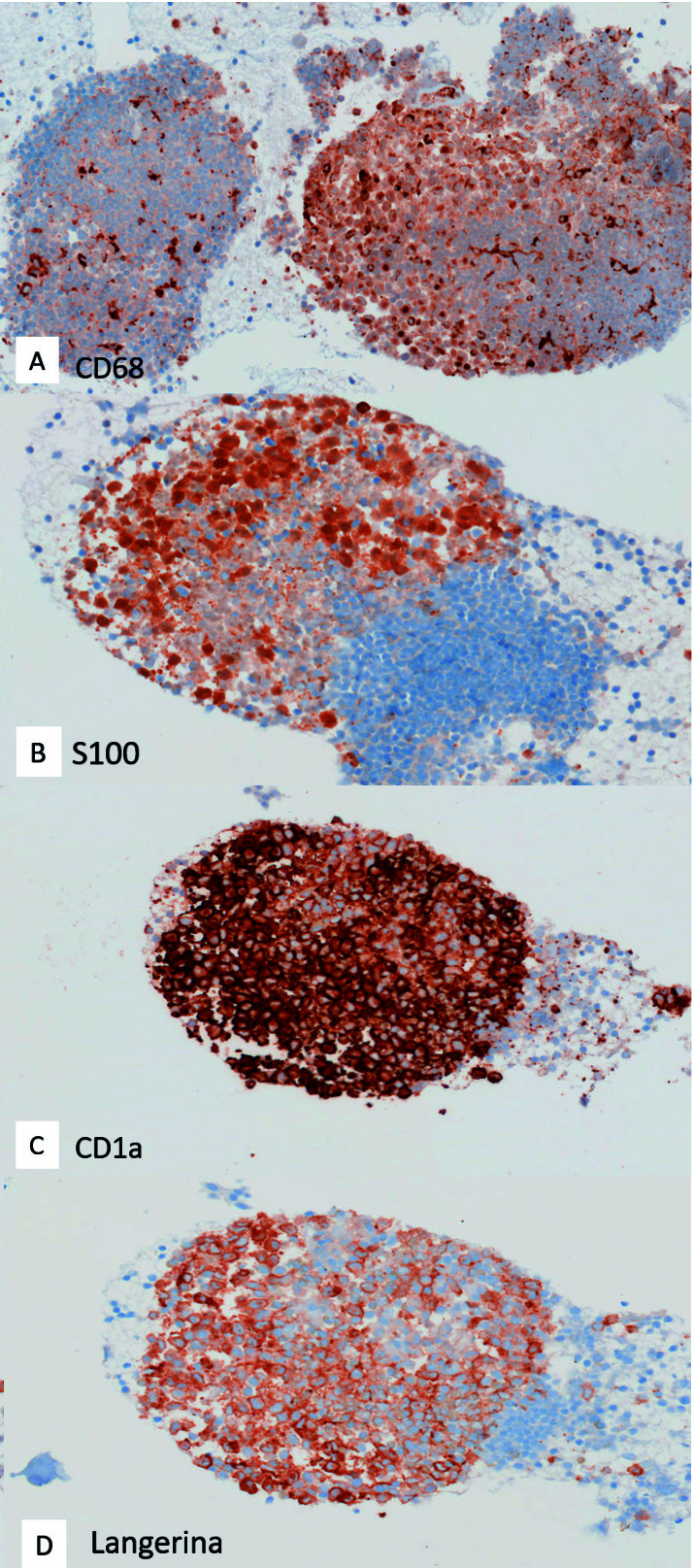
Estudio inmunohistoquímico. Inmunorreactividad citoplasmática para CD68 (A), S100 (B), CD1a (C) y Langerina (D) que confirma que se trata de células de Langerhans. 200x.

El estudio inmunohistoquímico mostró inmunorreactividad para CD68, CD1a, Langerina y S100 (Fig. 4).

El diagnóstico final por PAAF-EBUS de las adenopatías subcarinal y paratraqueal derecha fue negativo para malignidad, histiocitosis de células de Langerhans.

Tras el diagnóstico se decidió incluir al paciente en un programa intensivo de terapia cognitivo-conductual de deshabituación tabáquica y, por sus problemas articulares, continuar con prednisona 5mg/24h y Celecoxib 200 mg/24h.

## DISCUSIÓN

La histiocitosis de células de Langerhans es una enfermedad poco frecuente^1^ que consiste en la proliferación clonal de células de Langerhans con una morfología, perfil inmunofenotípico y ultraestructural caracterizado por expresar CD1a, Langerina y S100. En un porcentaje de casos (5-25%) la detección de afectación pulmonar o de ganglios mediastínicos es un hallazgo incidental en radiología de tórax de rutina en pacientes sin sintomatología previa relacionada^3^, como en este caso.

El manejo citológico de la muestra supone un reto por su pequeño tamaño y las dificultades para procesarla optimizando el rendimiento del material obtenido, de forma que posibilite realizar las técnicas complementarias requeridas para emitir un diagnóstico completo y preciso, descartando otras patologías y procesos relacionados. Hasta la fecha, los casos con afectación mediastínica diagnosticados mediante citología son muy escasos en la literatura^4^^-^^6^, la mayoría diagnosticados por lavado bronquioloalveolar como en el manuscrito de Gupta y col^6^^,^^7^. No se ha encontrado ningún caso diagnosticado mediante PAAF-EBUS.

Los hallazgos citológicos que observamos coinciden con los hallazgos de los casos publicados^1^. Son extensiones hipercelulares, con abundantes células de Langerhans. Estas células tienen núcleos son ovalados y polilobulados con un aspecto arriñonado. Las membranas nucleares son irregulares y muestran surcos, barras nucleares, pliegues e indentaciones. La cromatina es fina, y los nucléolos son poco marcados o *discretos*. El citoplasma de las células es moderado y ligeramente eosinofílico. Las células de Langerhans muestran inmunorreactividad citoplasmática para CD68, CD1a, Langerina (CD207) y S100. Entremezcladas con las células de Langerhans se observan algunos eosinófilos y células gigantes multinucleadas. No es frecuente, pero puede aparecer necrosis e infiltrado neutrofílico^2^^,^^6^. La atipia nuclear es mínima; sin embargo, la actividad mitótica es variable. Aunque las mitosis típicas pueden ser incluso abundantes, es infrecuente encontrar mitosis atípicas (explosivas o multipolares). No se acepta la abundancia de mitosis atípicas y, si estuvieran presentes, habría que plantear un diagnóstico de malignidad o proceso linfoproliferativo^4^.

En nuestro caso, el diagnóstico diferencial clínico incluía la sarcoidosis, entidad rápidamente descartada por carecer de granulomas no necrotizantes. De acuerdo con los hallazgos citológicos, el principal diagnóstico diferencial que tuvimos en cuenta fue la linfadenitis dermatopática, puesto que comparte con la HCL la presencia de células de Langerhans. Esta entidad se diferencia de la HCL por, principalmente, la localización de la afectación ganglionar, que suele ser en ingle y axilas, la existencia frecuente de antecedentes de afectación cutánea y, por último, histológicamente el infiltrado ganglionar suele ser de localización paracortical y carece de eosinófilos. Todos los diagnósticos diferenciales planteados se incluyen en la Tabla 1.

La HCL puede ser unifocal o multifocal (diseminada), siendo la forma unifocal la más frecuente y asociada con un mejor pronóstico, como en nuestro caso, mientras que la enfermedad multisistémica se asocia con mal pronóstico. En la forma unifocal, los órganos mayormente afectados son hueso o tejido blando adyacente (cráneo, mandíbula, fémur, vértebra, huesos pélvicos y costillas), siendo infrecuente la afectación de ganglios linfáticos (cervical, mediastino, inguinal, axilar o retroperitoneal), piel y pulmones^2^. Lo infrecuente de la presentación y la localización de difícil acceso dan valor a este caso que presentamos.

**Tabla 1:** Diagnóstico citológico diferencial de afectación mediastínica

Diagnósticos diferenciales	Características	Inmunohistoquímica
Sarcoidosis	Granulomas sarcoideos.	*Positividad*: CD68
Histiocitosis de células de Langerhans	Infiltrado sinusoidal de células de Langerhans y eosinófilos.	*Positividad*: CD1a, Langerina, S100
Linfadenitis dermatopática	Expansión paracortical con aumento de células dendríticas interdigitantes, células de Langerhans e histiocitos/macrófagos (que normalmente incluyen melanófagos)	*Positividad*: CD68, CD1a, Langerina, S100
	Ausencia de microabscesos eosinofílicos.	
Enfermedad de Kimura	Infiltrados eosinofílicos.	*Positividad*: Depósitos de IgE +
	Carece de una población significativa de células de Langerhans.	*Negatividad*: CD1a, Langerina, S100
Enfermedad de Rosai-Dorfman	Infiltrado histiocítico y linfoplamocítico.	Emperipolesis
		*Positividad*: Marcadores macrofágicos, S100
		*Negatividad*: CD1a, Langerina

Cerca de un 50% de los casos de HCL, tanto multifocal como unifocal, presentan la mutación del protooncogen *B-RAF* V600E; en un 35% de los casos en los que BRAF no está mutado, existen otras mutaciones activadoras de la vía RAS/MAPK^3^^,^^8^^,^^9^. Aunque las mutaciones no son necesarias para el diagnóstico, pueden suponer un avance en terapia dirigida^1^. En nuestro paciente se observó una intensa inmunorreactividad citoplasmática para BRAF V600E (Anexo I). A pesar de que la detección mediante secuenciación molecular es considerado el método *gold standard*, la inmunotinción de la proteína VE1 es una herramienta sensible y específica para la detección de mutaciones BRAF V600E^9^^,^^10^.

En casos de afectación pulmonar y/o mediastínica, el tratamiento consiste en eliminar el tabaquismo, que es el principal factor de riesgo asociado, como en el caso de nuestro paciente.

El caso presentado ilustra cómo es posible realizar un diagnóstico citológico mínimamante invasivo de la histiocitosis de células de Langerhans con afectación de ganglios mediastínicos mediante PAAF-EBUS. Un buen manejo del material por parte del citopatólogo presente en la sala puede ser clave para posibilitar un estudio completo del tejido que permita realizar un diagnóstico preciso, evitando abordajes diagnósticos más agresivos.

## Figures and Tables

**Figure:**
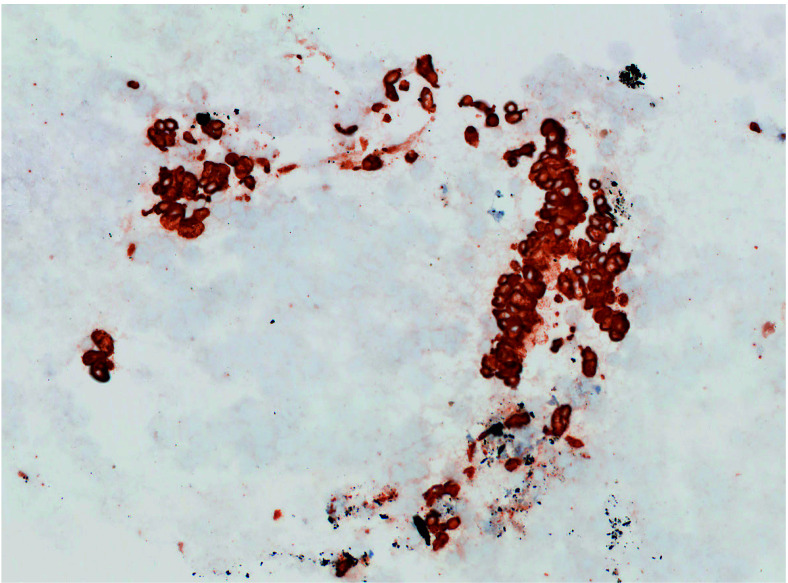
ESTUDIO INMUNOHISTOQUÍMICO. Las células de Langerhans muestran inmunotinción citoplasmática frente al anticuerpo monoclonal de ratón VENTANA anti-BRAF V600E (VE1).

## Data Availability

Se encuentran disponibles bajo petición a la autora de correspondencia.
